# Photosynthetic Responses of *Pontederia cordata* to Cadmium Stress: Anatomical Structure, Ultrastructure, Physiology, and Gene Expression

**DOI:** 10.3390/plants14091344

**Published:** 2025-04-29

**Authors:** Yan Li, Wei Zhou, Hanwen Xiao, Jianpan Xin, Chu Zhao, Runan Tian

**Affiliations:** College of Landscape Architecture, Nanjing Forestry University, Nanjing 210037, China; liyan1120@njfu.edu.cn (Y.L.);

**Keywords:** *Pontederia cordata*, cadmium, leaf structure characters, photosynthetic parameters, gene expression, carbon metabolism

## Abstract

*Pontederia cordata*, a horticulturally valuable ornamental plant, exhibits cadmium (Cd) tolerance, but its photosynthetic response to Cd stress has not been fully elucidated. Here, we employed hydroponics to investigate the effects of varying Cd concentrations on the leaf morphology, anatomy, photosynthetic physiology, and carbon metabolism enzymes in *P. cordata*. At 0.1 mM Cd, the plants grew well and showed no toxicity, with a normal chloroplast ultrastructure and chlorophyll *a* fluorescence parameters. Higher Cd concentrations (0.2–0.4 mM) disrupted chloroplasts, reduced chlorophyll content, and suppressed photosynthetic enzyme expression, thereby impairing light energy conversion efficiency and photosynthetic performance. In response, *P. cordata* adapted by maintaining the thickness of the palisade tissue, increasing the ratio of palisade tissue thickness to spongy tissue thickness, stabilizing carotenoid levels, enhancing non-photochemical quenching processes, and increasing the content of key photosynthetic enzymes and soluble sugars. These findings advance the theoretical understanding of photosynthetic adaptation mechanisms to heavy metal stress.

## 1. Introduction

Heavy metal pollution is a global environmental issue that poses severe threats to human health and ecosystem stability. Among the heavy metals, cadmium (Cd) is a primary concern because of its mobility, toxicity, and bioaccumulation properties, even at relatively low concentrations. The main sources of Cd in the environment are anthropogenic, including agricultural sources, such as fertilizers and pesticides, as well as industrial and mining activities [1]. Cd is highly phytotoxic and is readily absorbed by plant roots in soil and water, where it is subsequently transported to aboveground tissues via the xylem and phloem. Cd toxicity has been reported to affect plants at morphological, physiological, biochemical, and molecular levels [2].

Cd toxicity significantly impairs leaf structure and photosynthetic function, establishing them as reliable biomarkers for environmental stress responses. In healthy leaves, mesophyll cells maintain their structural integrity with spindle-shaped chloroplasts aligned along cell walls, facilitating optimal light capture and photosynthetic product transport. Photosynthesis is vulnerable to Cd interference, with direct and indirect effects on both light-dependent reactions and the Calvin cycle [3]. The photosynthetic membrane is extremely sensitive to Cd, with damage observed at multiple levels, including chloroplasts’ ultrastructure, pigments, proteins, and lipid composition [4]. Liang et al. [5] observed chloroplast deformation and reduced starch granules in *Oryza sativa* following 5 mg·kg^−1^ Cd exposure. Cd disrupts thylakoid electron transport through two main mechanisms, as follows: (1) replacement of Mn_4_CaO_5_ cluster cations in PSII’s oxygen-evolving complex, directly impairing PSII activity; and (2) inhibition of root iron (Fe) uptake, which compromises chloroplast Fe transport and ferredoxin synthesis, ultimately downregulating PSI activity [6]. Under Cd stress, plants activate defense mechanisms to safeguard their photosynthetic apparatus. Chlorophyll fluorescence, a sensitive and non-destructive method, reveals how plants respond by adjusting electron transport and related pathways [7].

In addition to its detrimental effects on the photosynthetic membrane and electron transport chain, Cd stress also disrupts the Calvin cycle. These reaction intermediates serve as important precursors in the biosynthesis of sucrose and starch. During the carbon fixation stage, ribulose-1,5-bisphosphate carboxylase/oxygenase (RuBisCO), the most crucial enzyme in the Calvin cycle, catalyzes the fixation of CO_2_ into ribulose-1,5-bisphosphate (RuBP), producing the initial photosynthetic product, 3-phosphoglycerate (3-PGA) [8,9]. During the carbon reduction stage, glyceraldehyde-3-phosphate dehydrogenase (GAPDH) catalyzes the reduction of 3-PGA to glyceraldehyde-3-phosphate (G3P). Cd stress reduced GAPDH activity in pepper seedling leaves, disrupting Calvin cycle enzymes and decreasing carbon assimilation efficiency [10]. During the regeneration of the RuBP substrate, a portion of G3P is utilized for the synthesis of sucrose and starch, while the remaining part ultimately generates RuBP through catalysis by enzymes, such as fructose-1,6-bisphosphatase (FBPase) [11,12]. Green plants contain two isoforms of FBPase, which play pivotal roles in sucrose metabolism and the CO_2_ reduction process in photosynthesis. Their activity directly influences carbohydrate accumulation and photosynthetic efficiency [13]. Mukherjee et al. [14] proposed that abiotic stress affects FBPase activity in rice seedlings. According to Siddiqui et al. [12], Cd stress significantly reduced FBPase activity in tomato seedlings, whereas exogenous potassium and melatonin increased it by approximately 2.5–3-fold, effectively regulating carbon partitioning and sugar metabolism.

Soluble sugars, particularly sucrose, glucose, and fructose, play pivotal roles in regulating various physiological processes, such as plant growth and development, stress responses, and biotic defense reactions [15]. Cd stress interferes with photosynthesis, thereby affecting the content and distribution of soluble sugars in plants. Studies have shown that Cd stress leads to an increase in total soluble sugar levels in such plants as *Vigna radiata* [7] and *Spinacia oleracea* [16]. Sucrose, which is the primary product of CO_2_ fixation during photosynthesis, requires FBPase participation during its synthesis. Sucrose synthase (SUS) and sucrose phosphate synthase (SPS) are essential for plant sucrose metabolism [17]. SPS is highly active in photosynthetic tissues and serves as a critical regulator in the partitioning of photosynthetic products into sucrose and starch. Overexpression of the gene *CsSPS4* from *Cucumis sativus* in tobacco significantly increases the sucrose content, sucrose-to-starch ratio, and leaf yield in transgenic plants [18].

While the photosynthetic responses to Cd stress have been extensively studied in model plants and crops, little is known about aquatic ornamental species. *Pontederia cordata*, a Cd-tolerant emergent aquatic plant, holds promise for phytoremediation due to its ecological and ornamental value [19]. We hypothesize that *P. cordata* employs unique structural and physiological adaptations to maintain photosynthetic function under Cd stress, distinct from terrestrial plants. To test this, we investigated the following topics: (1) morpho-anatomical adjustments in leaves and chloroplasts, (2) photosynthetic efficiency through pigment content, gas exchange parameters, and chlorophyll fluorescence, and (3) photosynthetic carbon metabolism regulation via soluble sugar content, sucrose metabolism, and key enzyme expression. This study provides the first comprehensive analysis of Cd-induced photosynthetic adaptations in *P. cordata*, establishing a theoretical foundation for selecting tolerant plants and advancing phytoremediation strategies for heavy metal-contaminated aquatic systems.

## 2. Results

### 2.1. Morphological Changes in Pontederia cordata Under Cd Treatment

*P. cordata* in the CK and T1 exhibited no visible toxic symptoms and maintained healthy growth at 7 and 14 d after treatment, with a damage level of 0 (Figure S1 and Table S2). Compared to the CK, the damage level of the plants in the Cd treatments increased with increasing Cd concentrations. At 14 d, plants in T4 showed the most severe leaf toxicity, with a damage level of four, characterized by extensive leaf withering, defoliation, and even plant death, as well as stem softening, chlorosis, and drying. These results indicated that Cd stress inhibited the growth of *P. cordata*.

### 2.2. Changes in Leaf Anatomical Structure Under Cd Treatment

**Figure 1:**
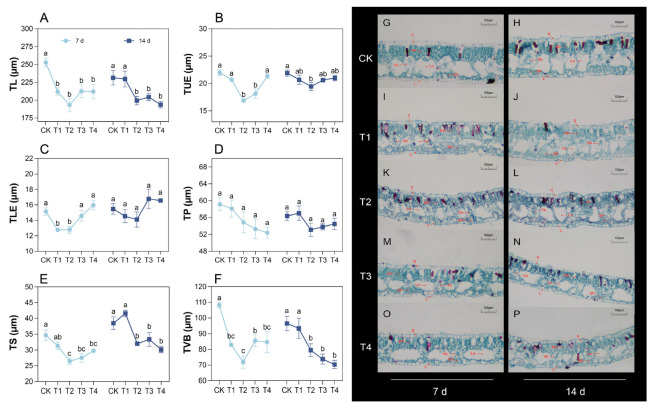
Changes in leaf anatomical structure of *P. cordata* after 7 d and 14 d exposure to 0 mM (CK), 0.1 mM (T1), 0.2 mM (T2), 0.3 mM (T3), and 0.4 mM (T4) Cd^2+^. (**A**–**F**) Quantitative changes in leaf anatomical parameters, including TL (total leaf thickness), TUE (upper epidermis thickness), TLE (lower epidermis thickness), TP (palisade tissue thickness), TS (spongy tissue thickness), and TVB (vascular bundle thickness). (**G**–**P**) Transverse sections of *P. cordata* leaves showing structural features. Anatomical labels: U (upper epidermis), L (lower epidermis), PP (palisade tissue), SP (spongy tissue), Vb (vascular bundle), and La (lumen). Data represent mean ± SE (*n* = 3). Different lowercase letters per day indicate significant differences among treatments (one-way ANOVA, Tukey’s test, *p* ≤ 0.05).

Compared with the CK group, the total leaf thickness (TL) of *P. cordata* significantly decreased by 15.93–23.59% in T1–T4 at 7 d after treatment and by 11.72–16.27% in T2–T4 at 14 d (Figure 1A). The minimum of upper epidermis thickness (TUE) was observed in T2 (Figure 1B). At 7 d, the lower epidermis thickness (TLE) initially decreased and then increased with increasing Cd concentration (Figure 1C). On day 14, no significant differences in TLE were observed among the groups (*p* > 0.05). Cd treatment had no significant effect on the palisade tissue thickness (TP) (Figure 1D). Compared to the CK, the spongy tissue thickness (TS) in T2–T4 decreased by 14.22–23.78% and 13.25–21.60% at 7 and 14 d, respectively (Figure 1E). At 7 d, the change in the vascular bundle thickness (TVB) was generally consistent with that of the TL (Figure 1F). At 14 d, the TVB in T2–T4 was significantly reduced by 17.64–27.11% compared with the control.

### 2.3. Changes in Chloroplast Ultrastructure Under Cd Treatment

**Figure 2:**
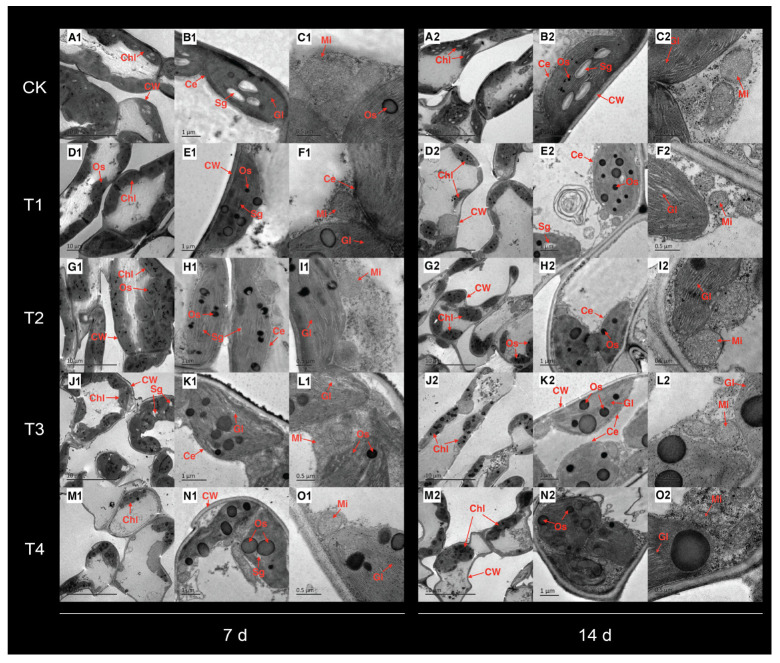
Transmission electron micrographs of chloroplast ultrastructure of leaves in *P. cordata* under (**A**–**C**) 0 mM (CK), (**D**–**F**) 0.1 mM (T1), (**G**–**I**) 0.2 mM (T2), (**J**–**L**) 0.3 mM (T3), and (**M**–**O**) 0.4 mM (T4) Cd^2+^ for 7 d (left panels) and 14 d (right panels). CW: cell wall, Chl: chloroplast, Gl: grana lamellae, Sg: starch granule, Os: osmiophilic granules, and Mi: mitochondrion.

At 7 d after treatment, the mesophyll cell structure of the CK remained intact, with chloroplasts appearing oval or ovoid and closely appressed to the cell membrane (Figure 2). The thylakoids were tightly packed with clear lamellar structures, and the mitochondria exhibited full morphology. In T1 and T2, chloroplasts were slightly swollen, osmiophilic granules began to increase, starch grains decreased, and mitochondria began to shrink. In T3 and T4, some chloroplasts underwent partial degradation and separated from the cell wall, with the double membrane beginning to dissolve. Osmiophilic granules became significantly larger and more abundant, starch grains markedly decreased, grana lamellae expanded with vacuoles appearing, and the mitochondria continued to shrink.

Fourteen days after treatment, the chloroplast ultrastructure of the CK and T1 exhibited no significant changes compared to that on the seventh day (Figure 2). In T2, mesophyll cells began to wrinkle, with a small portion of the chloroplasts separating from the cell wall. Osmiophilic granules increased in size and number, whereas starch grains decreased significantly. In T3, the double membrane started to dissolve, osmiophilic granules were further enlarged, grana lamellae expanded with small vacuoles, and mitochondria continued to shrink. In T4, mesophyll cells exhibited a disordered shape with pronounced wrinkling. Most chloroplasts were clearly separated from the cell wall, the double membrane was further dissolved, osmiophilic granules increased significantly in size and number, starch grains were no longer observable, the grana lamellae expanded with vacuoles, and some mitochondria became elliptical without complete disintegration.

### 2.4. Changes in Photosynthetic Pigment Content Under Cd Treatment

**Figure 3:**
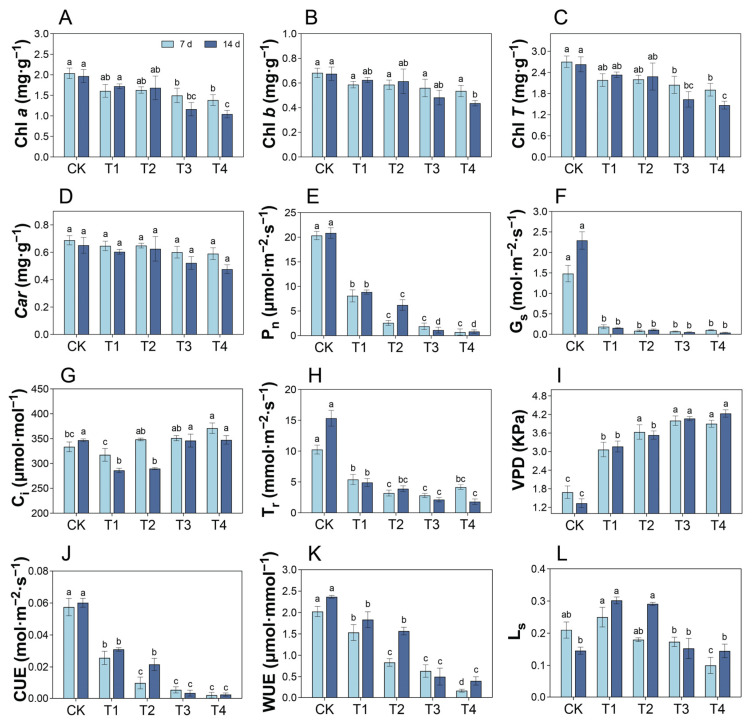
Changes in photosynthetic pigment content and photosynthetic gas exchange of leaves in *P. cordata* after 7 d and 14 d exposure to 0 mM (CK), 0.1 mM (T1), 0.2 mM (T2), 0.3 mM (T3), and 0.4 mM (T4) Cd^2+^. Data represent mean ± SE (*n* = 3). Different lowercase letters per day indicate significant differences among treatments (one-way ANOVA, Tukey’s test, *p* ≤ 0.05).

After 7 and 14 d of treatment, compared with the CK, the Chl *a* content of *P. cordata* leaves in T1 and T2 showed no significant changes (*p* > 0.05), whereas those in T3 and T4 were significantly reduced (Figure 3A,C). The trend in Chl *T* content was consistent with that of Chl *a*. The 7 d Cd treatment had no significant effect on the Chl *b* contents in the leaves of all groups, whereas at 14 d, the Chl *b* content in T4 decreased by 35.29% compared with that in CK (Figure 3B). Treatment for 7 and 14 d had no significant inhibitory effect on the *Car* content in the leaves (Figure 3D).

### 2.5. Changes in Photosynthetic Gas Exchange Under Cd Treatment

Compared with the CK, the P_n_ in the leaves of *P. cordata* decreased by 60.22–96.41% and 57.50–96.02% after 7 and 14 d of treatment, respectively (Figure 3E). In addition, the trend of leaf CUE was consistent with that of P_n_ (Figure 3J). Compared with CK, leaf C_i_ significantly increased by 11.30% in T4 7 d after treatment, whereas it decreased by 17.50% and 16.44% in T1 and T2 at 14 d, respectively (Figure 3G). The G_s_, T_r_, and WUE were significantly inhibited after 7 and 14 d (Figure 3F,H,K). However, the VPD significantly increased after 7 and 14 d (Figure 3I). Moreover, only T4 had a significant decrease in leaf L_s_ at 7 d compared to the CK, whereas the L_s_ of T1 and T2 increased significantly at 14 d (Figure 3L).

### 2.6. Changes in Leaf Chlorophyll a Fluorescence Parameter Under Cd Treatment

**Figure 4:**
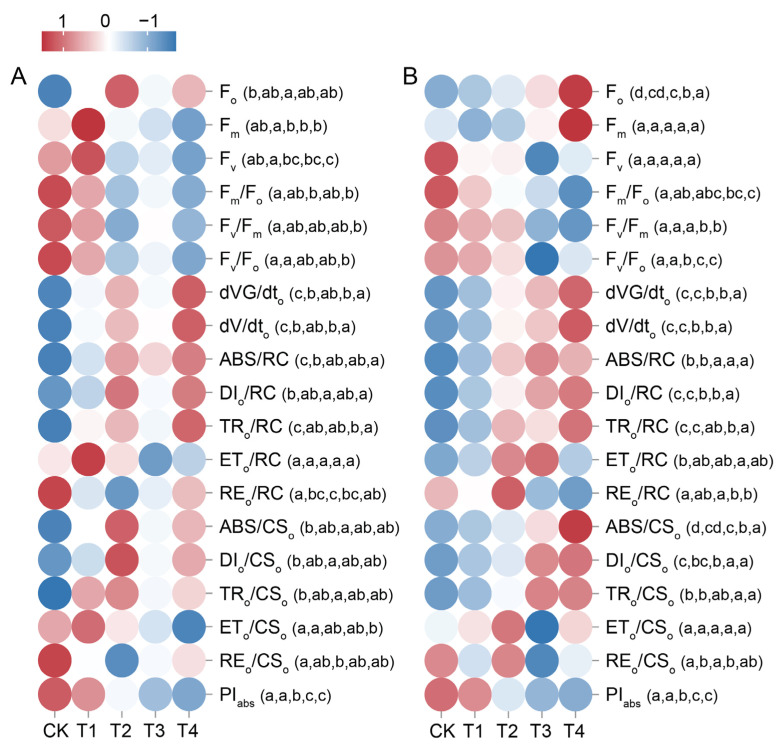
Heatmaps displaying changes in chlorophyll *a* fluorescence parameter of leaves in *P. cordata* exposed to 0 mM (CK), 0.1 mM (T1), 0.2 mM (T2), 0.3 mM (T3), and 0.4 mM (T4) Cd^2+^ for 7 d (**A**) and 14 d (**B**). Color scale indicates normalized values (red: high; blue: low). Different lowercase letters per row indicate significant differences among treatments (one-way ANOVA, Tukey’s test, *p* ≤ 0.05).

After 7 days of treatment, the *F*_o_, DI_o_/RC, ABS/CS_o_, DI_o_/CS_o_, and TR_o_/CS_o_ of *P. cordata* leaves peaked in T2, increasing by 48.05%, 143.75%, 48.05%, 146.45%, and 23.29%, respectively, compared with the CK (Figure 4A and Table S3). However, the RE_o_/RC and RE_o_/CS_o_ reached their lowest values at T2, decreasing by 43.60% and 39.26%, respectively. Compared with the CK, the *F*_v_, *F*_m_/*F*_o_, *F*_v_/*F*_m_, *F*_v_/*F*_o_, and ET_o_/CS_o_ of the leaves significantly decreased in T4 by 23.04%, 34.74%, 13.75%, 43.47%, and 23.43%, respectively. Meanwhile, 0.1–0.4 mM Cd treatment for 7 d had no significant effect on leaf *F*_m_ and ET_o_/RC (*p* > 0.05). However, the above Cd concentrations significantly increased the leaf dVG/dt_o_, dV/dt_o_, ABS/RC, and TR_o_/RC. In addition, the PI_abs_ of T2–T4 significantly reduced by 48.64–81.81% compared to the CK group and were significantly lower than that of T1.

Compared with the CK, the *F*_o_, dVG/dt_o_, dV/dt_o_, ABS/RC, DI_o_/RC, TR_o_/RC, ABS/CS_o_, and DI_o_/CS_o_ of the leaves significantly increased in T2–T4 14 d after treatment, whereas *F*_v_/*F*_o_ and PI_abs_ significantly decreased (Figure 4B and Table S3). Fourteen days of Cd treatment at the above-mentioned concentrations had no significant effect on leaf *F*_m_, *F*_v_, or ET_o_/CS_o_ (*p* > 0.05). Compared with the CK group, the *F*_m_/*F*_o_, *F*_v_/*F*_m_, and RE_o_/RC in T1 and T2 showed no significant changes (*p* > 0.05), whereas they significantly decreased in T3 and T4.

### 2.7. Changes in Leaf Soluble Sugar Content Under Cd Treatment

**Figure 5:**
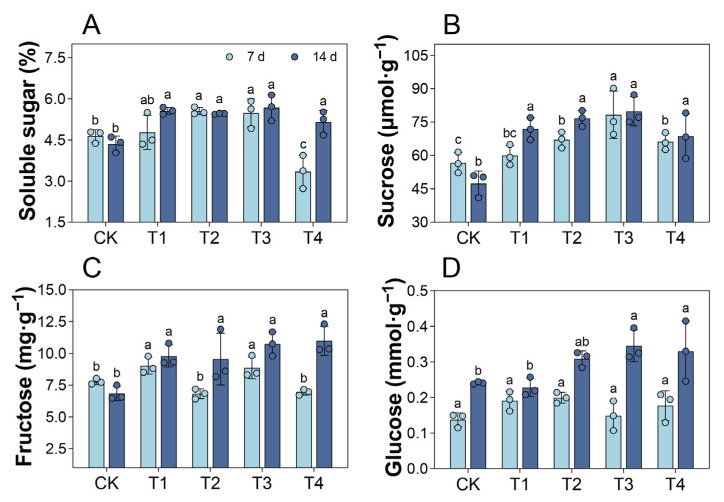
Soluble sugar accumulation of leaves in *P. cordata* after 7 d and 14 d exposure to 0 mM (CK), 0.1 mM (T1), 0.2 mM (T2), 0.3 mM (T3), and 0.4 mM (T4) Cd^2+^: (**A**) soluble sugar, (**B**) sucrose, (**C**) fructose, and (**D**) glucose. Data represent mean ± SE (*n* = 3). Different lowercase letters per day indicate significant differences among treatments (one-way ANOVA, Tukey’s test, *p* ≤ 0.05).

Compared with the CK, the soluble sugar content of the leaves in T1 showed no significant change 7 d after treatment, whereas it significantly increased in T2 and T3 and significantly decreased in T4 (Figure 5A). At 14 d, the soluble sugar content significantly increased by 18.39–30.57% in T1–T4. Compared with the CK, only T1 showed no significant increase in sucrose content at 7 d (*p* > 0.05); however, fourteen days of treatment with 0.1–0.4 mM Cd resulted in a significant increase in sucrose content of 44.88–68.41% (Figure 5B). Fourteen days of treatment with these Cd concentrations also led to a significant increase in fructose content in the leaves (Figure 5C). Cd treatment for 7 d had no significant effect on the glucose content (Figure 5D). However, at 14 d, the glucose content in T1 and T2 showed no significant change compared to the CK (*p* > 0.05), whereas it significantly increased in T3 and T4.

### 2.8. Changes in Leaf SUS and SPS Activities and Their Gene Expression Under Cd Treatment

**Figure 6:**
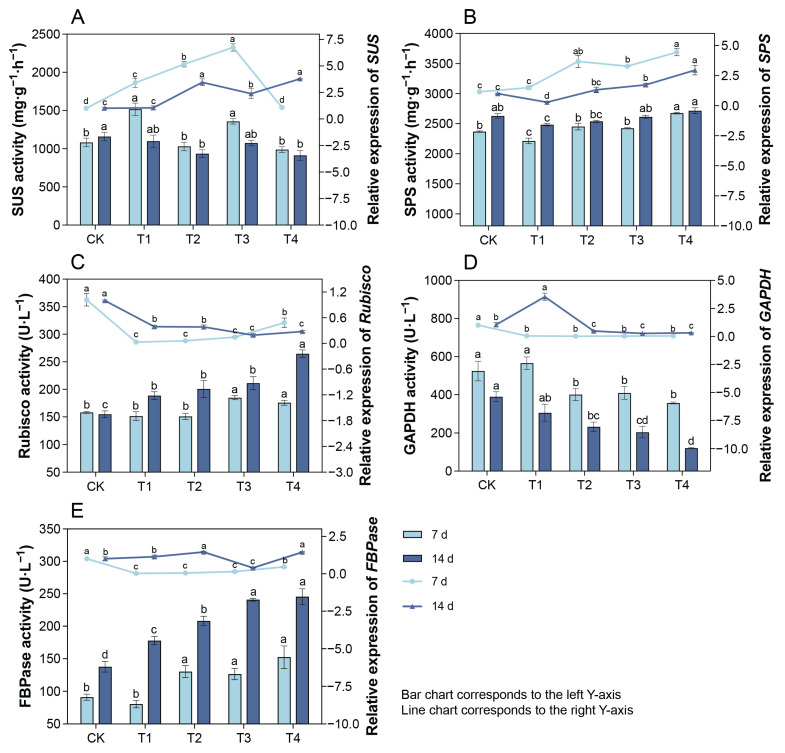
Enzyme activities and gene expression of (**A**) SUS, (**B**) SPS, (**C**) RuBisCO, (**D**) GAPDH, and (**E**) FBPase of leaves in *P. cordata* after 7 d and 14 d exposure to 0 mM (CK), 0.1 mM (T1), 0.2 mM (T2), 0.3 mM (T3), and 0.4 mM (T4) Cd^2+^. Data represent mean ± SE (*n* = 3). Different lowercase letters per day indicate significant differences among treatments (one-way ANOVA, Tukey’s test, *p* ≤ 0.05).

Seven days after treatment, the SUS activities in the leaves of T1 and T3 were significantly higher than that of the CK group; *SUS* expressions in T1–T3 exhibited upward trends, while that of T4 returned to the control level (Figure 6A). At 14 d, the SUS activities of T2 and T4 were significantly lower than that of the CK, whereas *SUS* expressions in T2–T4 were significantly higher than that of the CK. Seven days after treatment, SPS activity in the leaves of T4 was significantly higher than that in the CK, and *SPS* expressions in T2–T4 were significantly higher than that in the CK (Figure 6B). At day 14, both the activity and relative expression level of SPS showed trends of first decreasing and then increasing.

### 2.9. Changes in Leaf RuBisCO, GAPDH, and FBPase Activities and Their Gene Expression Under Cd Treatment

Compared with the CK, the RuBisCO activities in the leaves of T3 and T4 significantly increased 7 d after treatment, whereas the relative expression levels of *RuBisCO* in T1–T4 significantly decreased (Figure 6C). After 14 d, the RuBisCO activity and relative expression level exhibited increasing and decreasing trends, respectively. The activities and relative expression levels of GAPDH in the leaves of T2–T4 were significantly lower than those of the CK (Figure 6D). At 7 d, the FBPase activities in the leaves of T2–T4 significantly increased compared with that in the CK, whereas *FBPase* expression significantly decreased (Figure 6E). At 14 d, the variation trend of the FBPase activity was consistent with that observed at 7 d. Compared with the CK, the *FBPase* expression levels significantly increased in T2 and T4 but significantly decreased in T3.

### 2.10. Correlation Analysis of Leaf Photosynthetic Physiological Indices Under Cd Treatment

**Figure 7:**
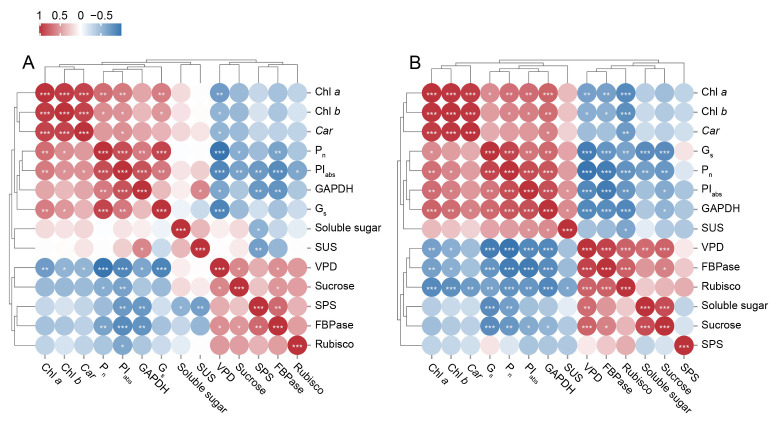
Correlation maps of photosynthetic physiological parameters, soluble sugar, and relevant enzymes for 7 d (**A**) and 14 d (**B**). * Correlation was statistically significant at a *p*-value between 0.01 and 0.05. ** Correlation is statistically significant at a *p*-value between 0.001 and 0.01, and *** Correlation was statistically significant at the *p* < 0.001 level for the Pearson correlation coefficient.

At both 7 and 14 d of treatment, Chl *a* and Chl *b* contents in *P. cordata* leaves were positively correlated with P_n_ and PI_abs_ (Figure 7). This indicated that a decrease in chlorophyll content may be an important physiological factor contributing to reduced photosynthetic efficiency under Cd stress. Additionally, both P_n_ and PI_abs_ were positively correlated with GAPDH and G_s_ but negatively correlated with VPD, sucrose content, and FBPase content. Furthermore, sucrose content was positively correlated with FBPase activity, which was attributed to the involvement of FBPase in sucrose synthesis.

## 3. Discussion

A plant’s structure is the foundation for its function, and changes in plant structure inevitably lead to alterations in physiological and ecological functions. Leaves serve as material carriers and vital organs for photosynthesis in plants. Many plant leaves consist of two photosynthetic tissue layers, namely the palisade and spongy mesophylls, of which the palisade mesophyll has a higher photosynthetic capacity [20]. In this study, we observed that, after treatment with 0.1 mM Cd, the thicknesses of the palisade and spongy tissues in *P. cordata* leaves did not significantly differ from those in the control. However, after treatment with 0.2–0.4 mM Cd, the thickness of the palisade tissue remained unchanged, while that of the spongy tissue significantly decreased. This indicated that maintaining the thickness of the palisade tissue and increasing the ratio of palisade tissue thickness to spongy tissue thickness represent the adaptive mechanisms of *P. cordata* to Cd stress. This is beneficial for maintaining effective CO_2_ absorption and diffusion within the leaves, adjusting chlorophyll distribution, and optimizing photosynthesis [21,22]. Compared with the analysis of leaf anatomical structure, electron microscopy image analysis, which compares the damage to the plant ultrastructure caused by varying degrees of stress, can more accurately distinguish between the adaptive and stressed states of plants under adverse conditions. An intact chloroplast structure is a prerequisite for normal plant photosynthesis. In this study, chloroplasts in *P. cordata* leaves maintained a normal morphology after treatment with 0.1 mM Cd. However, after treatment with 0.2–0.4 mM Cd, chloroplasts in the leaves gradually deformed, with the double membrane and starch grains dissolving, grana lamellae loosening, and osmiophilic granules increasing. These changes are indicative of stress-induced damage and are consistent with the responses of *Morus alba* [23], *Salvia sclarea* [5], and barley [24] to Cd stress. This suggested a decline in the photosynthetic capacity of *P. cordata* leaves under 0.2–0.4 mM Cd stress, as manifested by a reduction in P_n_.

Beyond structural alterations, the stability of photosynthetic pigments—especially chlorophyll—serves as another key determinant of photosynthetic efficiency. Chlorophyll not only gives plant leaves their green color but also plays a pivotal role in photosynthesis by absorbing light energy for conversion into chemical energy, thereby driving the energy transformations required for plant growth and development. The content and status of chlorophyll directly affect the rate of photosynthesis. In this study, after treatment with 0.1–0.2 mM Cd, no significant changes were observed in the Chl *a* and Chl *T* contents of *P. cordata* leaves compared with the CK. However, after treatment with 0.3–0.4 mM Cd, the Chl *a* and Chl *T* contents significantly decreased, accompanied by leaf chlorosis (Figure S1). This indicated that high Cd concentrations inhibited the biosynthesis of chlorophyll in *P. cordata* leaves, likely due to Cd (1) damaging chloroplast structure; (2) suppressing the expression of key enzymes involved in chlorophyll synthesis, such as glutamyl-tRNA reductase, delta-aminolevulinic acid dehydratase, porphobilinogen deaminase, uroporphyrinogen-III synthase, protoporphyrinogen oxidase, magnesium-chelatase subunit ChlH, NADPH-protochlorophyllide oxidoreductase, and chlorophyll synthase [25]; (3) binding to the thiol groups of enzymes, such as protochlorophyllide reductase, δ-aminolevulinic acid synthase, and 5-aminolevulinic acid dehydratase, thereby altering their normal configurations and, thus, inhibiting their activities; (4) inhibiting the uptake of magnesium, nitrogen, and iron; and (5) preventing the chelation of Mg^2+^ with protoporphyrin IX [26]. In photosynthesis, carotenoids (*Car*) are antenna pigments that transfer captured light energy to chlorophyll. They also play a role in scavenging reactive oxygen species (ROS) and protecting chlorophyll molecules from photooxidative damage. In this study, Cd stress did not cause significant changes in the *Car* content of *P. cordata* leaves, which was consistent with previous observations in tobacco [25]. This suggested that maintaining stable carotenoid levels is an adaptive mechanism of *P. cordata* to Cd stress.

While pigment stability ensures light absorption capacity, the actual photosynthetic performance is ultimately reflected in gas exchange dynamics. Photosynthetic gas exchange parameters are crucial indicators of the photosynthetic physiological status of plants under heavy metal stress. Studies have shown that stomatal and non-stomatal limitations represent two distinct mechanisms that describe the reduction in the photosynthetic rate [27]. In this study, after 14 d of treatment with 0.1–0.2 mM Cd, the decrease in G_s_ was accompanied by a reduction in *C*_i_ and an increase in L_s_, indicating that stomatal factors dominated the reduction in P_n_ in *P. cordata* leaves. Following treatment with higher Cd concentrations (0.3–0.4 mM), the P_n_ and G_s_ were significantly reduced compared to the CK, while Ci did not decrease. This suggested that non-stomatal factors, such as chloroplast structural damage, decreased chlorophyll content, and reduced expression and activity of GAPDH, predominantly limit P_n_ [1]. At this point, the fixation and utilization of CO_2_ by *P. cordata* leaves were reduced and the photosynthetic capacity was impaired, as evidenced by reductions in CUE and WUE [9]. Additionally, under Cd stress, VPD exhibited a significant negative correlation with G_s_ (day 7: y = −853.43 x + 3282, R^2^ = 0.8617; day 14: y = −791.28 x + 3107.4, R^2^ = 0.8978), and T_r_ significantly decreased compared to the CK. This indicated that under the Cd treatment, the G_s_ of *P. cordata* decreased with increasing VPD, thereby hindering transpiration and ultimately leading to imbalanced leaf water metabolism, manifested as leaf desiccation (Figure S1). Similar results were observed in *Pistia stratiotes* under Cd stress [28].

Complementary to gas exchange measurements, chlorophyll fluorescence analysis provides deeper insights into the photochemical processes underlying these photosynthetic responses. Chlorophyll fluorescence, a sensitive probe for plant photosynthesis, is widely used in photosynthetic physiology research and detection analysis of abiotic stress [29]. In this study, chlorophyll *a* fluorescence parameters were employed to elucidate the effect of Cd stress on the photosynthetic apparatus (primarily PSII) in *P. cordata* leaves, as well as the adaptive mechanisms of the photosynthetic system in response to Cd stress. F_o_ represents the fluorescence yield when the PSII reaction centers are fully open, and an increase in its value indicates that the plant is under stress. F_m_/F_o_ reflects the light energy absorption and electron transfer efficiency of the PSII reaction centers, where F_v_/F_m_ is the maximum photochemical quantum yield of PSII, which typically ranges from 0.8 to 0.85. The F_v_/F_o_ ratio indicates the potential activity of PSII and is commonly used to assess photosynthetic efficiency [30]. After treatment with 0.1 mM Cd, no significant changes were observed in F_o_, F_m_/F_o_, F_v_/F_m_, and F_v_/F_o_ in *P. cordata* leaves compared to those in the CK, indicating that the structure and function of PSII were not significantly affected. However, after 14 d of treatment with 0.2–0.4 mM Cd, the patterns of change in F_m_/F_o_, F_v_/F_m_, and F_v_/F_o_ consistently decreased, while F_o_ increased. These results indicated that treatment with 0.2–0.4 mM Cd caused a decline in the light energy conversion efficiency and photosynthetic performance of PSII reaction centers in *P. cordata* leaves, which was consistent with the decrease in RE_o_/RC and PI_abs_. As demonstrated by Waheed et al. [7], this suggested that the PSII reaction centers in *P. cordata* leaves experienced varying degrees of photoinhibition. This is associated with damage to the chloroplast ultrastructure, reduced Chl *a* content, impaired photosynthetic electron transfer [25], imbalances in ROS production and scavenging, and damage to the D1 protein [31]. The damage caused by photoinhibition and the plant’s own defense processes, including increasing the content of key photosynthetic enzymes, such as RuBisCO and FBPase, occurred simultaneously. In this study, after 14 d of treatment with 0.2–0.4 mM Cd, the absorbed light energy per reaction center and per excited-state area (ABS/RC and ABS/CS_o_) in *P. cordata* leaves increased, as did the energy captured by PSII reaction centers for reducing Q_A_ (TR_o_/RC and TR_o_/CS_o_). However, the energy entering the electron transport chain (ET_o_/RC and ET_o_/CS_o_) did not increase, whereas the energy dissipated as heat (DI_o_/RC and DI_o_/CS_o_) did increase. This is consistent with the findings of Li et al. [28], indicating that *P. cordata* leaves enhanced non-photochemical quenching processes to mitigate damage to the photosynthetic apparatus (including damage to the D1 protein and chlorophyll degradation) caused by excess excited energy (forming ROS) under Cd stress. This represents a photoprotective mechanism in the photosynthetic apparatus of *P. cordata* in response to high Cd concentrations stress [25].

In addition to protecting its photosynthetic apparatus, plants also employ metabolic adjustments through carbon metabolism to cope with heavy metal stress. Soluble sugars, such as sucrose, glucose, and fructose, function as osmotic regulators, energy sources, and signaling molecules, helping plants resist heavy metal stress and maintain normal growth and development [32]. In this study, after 14 d of treatment with 0.2–0.4 mM Cd, the contents of sucrose, glucose, fructose, and total soluble sugars in *P. cordata* leaves increased to varying degrees, consistent with observations in potato under Cd stress [33]. This indicated that increasing soluble sugar content is an adaptive mechanism of *P. cordata* to Cd stress. In the photosynthetic tissues of plants, sucrose synthesis occurs primarily via intermediates produced during photosynthesis. In sink tissues, sucrose is hydrolyzed into hexoses (glucose and fructose) or their nucleotide derivatives (UDP/ADP-glucose and fructose) through the enzymatic actions of invertase and SUS, respectively, facilitating its participation in various metabolic processes [34]. SUS is a glycosyltransferase that mediates the reversible conversion of sucrose and UDP (or ADP) into fructose and UDPG (or ADPG) [35]. Additionally, SPS is a key enzyme in sucrose synthesis, and overexpression of the SPS gene increases sucrose content in plants, such as sugarcane [17,36]. In this study, compared with T1, the relative expressions and activities of SUS in T3 and T4 exhibited dynamic changes, whereas those of SPS showed an upward trend. These results suggested that under 0.3–0.4 mM Cd conditions, *P. cordata* primarily promoted sucrose synthesis by increasing SPS gene expression and activity. Moreover, the accumulation of sucrose synthase and the corresponding soluble sugars implied a high energy demand of *P. cordata* during Cd stress and its alleviation [35].

Notably, divergent trends were observed between the mRNA levels and enzymatic activities for key photosynthetic proteins (e.g., RuBisCO, FBPase) across Cd^2+^ stress gradients (0–0.4 mM). This mismatch could be attributed to post-transcriptional and translational regulatory mechanisms. While mRNA levels reflect transcriptional activity, they inadequately predict functional enzyme capacity because translation efficiency and protein stability significantly influence final protein levels [37]. Li et al. [38] demonstrated that the activity of RuBisCO can be influenced by the redox state of cellular glutathione, which affects the translation of *RBCL* mRNA. This suggests that under Cd stress, the plant may prioritize maintaining RuBisCO or FBPase activities through post-translational modifications rather than through changes in mRNA levels. The observed mismatch between mRNA and protein levels underscores the importance of considering multiple layers of regulation when interpreting gene expression data.

## 4. Materials and Methods

### 4.1. Experimental Materials

Two-year-old *Pontederia cordata* plants were purchased from the Shengyue Flower and Seedling Nursery in Shuyang County, Jiangsu Province, China. The experiment was conducted in the greenhouse of the National Landscape Architecture Experimental Teaching Demonstration Center, Nanjing Forestry University, Nanjing, China (118°49′ E, 32°04′ N). Selected plants with good growth and a uniform size (approximately 50 cm in height) were initially cultivated in tap water for seven days. After rinsing the roots with ultrapure water, the plants underwent a 14-day adaptive cultivation period in 1/2 Hoagland nutrient solution, which was renewed every seven days.

### 4.2. Experimental Design

After adaptive cultivation, plants were subjected to a 14-day Cd stress treatment in a hydroponic system. The treatment solutions were prepared by dissolving CdCl_2_·2.5 H_2_O in 1/2 Hoagland nutrient solution. Based on previous research, the Cd^2+^ concentrations in the treatment groups were set at the following four gradients: 0.1 mM (T1), 0.2 mM (T2), 0.3 mM (T3), and 0.4 mM (T4). The control group (CK) received only 1/2 Hoagland nutrient solution. The aforementioned *P. cordata* plants were placed in turnover boxes (60 × 40 × 20 cm) containing the treatment solutions, with a 12-hole foam board laid to secure the plants. Each treatment consisted of 12 plants with 3 replicates. The day of treatment initiation was recorded as day 0, and the experiment lasted 14 days. Fresh leaf samples were collected on days 7 and 14 for physiological index measurements and gene expression experiments. All sample collections were conducted between 9:00–11:30 am to minimize diurnal variation effects.

### 4.3. Observation of Leaf Structure Characters in P. cordata

#### 4.3.1. Leaf Anatomical Structure

The second to third leaves from the top of *P. cordata* plants were collected, and 1 cm sections of lateral vein tissues were fixed in FAA fixative. Five distinct leaf tissue samples were collected from each replicate. The plant materials were subjected to dehydration in five different concentrations of ethanol, followed by embedding in paraffin at 60 °C. Conventional paraffin sectioning was performed by using a rotary microtome. Sections were stained with safranin fast green, cleared in xylene, and mounted with neutral gum [39]. The anatomical structures of the leaves were observed and photographed using an optical microscope.

#### 4.3.2. Chloroplast Ultrastructure

Lateral vein tissues (1–2 mm) were excised and immediately immersed in a fixative solution containing 2.5% glutaraldehyde and 2% paraformaldehyde, followed by overnight treatment at 4 °C. The tissues were then washed three times in 0.1 M phosphate buffer (pH 7.2) and fixed in 1% osmium tetroxide for 6 h. Subsequently, the tissues were dehydrated in a graded series of acetone solutions. After embedding in Spurr’s resin, the tissues were placed in EP tubes and processed in an oven at 60 °C for 48 h. Ultrathin sections (80 nm) were cut using an ultramicrotome and collected on nickel grids. Sections were stained with uranyl acetate in the dark for 15 min, washed with double-distilled water, and stained with lead citrate in the dark for 15–20 min. After washing and drying, chloroplast structures were observed and photographed using a transmission electron microscope [40]. Five fields of view were selected for observation per section.

### 4.4. Measurement of Photosynthetic Physiological Parameters

#### 4.4.1. Photosynthetic Pigment Content

The method described by Qiu et al. [41] was used with minor modifications. A 0.2 g sample of the leaf was placed in a mixture of acetone and ethanol (20 mL, in equal volumes). The samples were soaked in the dark for 24 h with occasional shaking to homogenize the extract until the tissue turned white and no green color remained. Using the acetone–ethanol mixture as a blank control, the absorbance values of the extracts were measured at the wavelengths of 663, 646, and 470 nm using a UV–Visible Spectrophotometer (Lambda 365, PerkinElmer, Waltham, MA, USA). Chlorophyll *a* (Chl *a*), chlorophyll *b* (Chl *b*), total chlorophyll (Chl *T*), and carotenoid (*Car*) contents were calculated as follows: Chl *a* (mg·g^−1^) = (12.21 × OD_663_ − 2.81 × OD_646_) × V/w, Chl *b* (mg·g^−1^) = (20.13 × OD_646_ − 5.03 × OD_663_) × V/w, Chl *T* (mg·g^−1^) = (17.32 × OD_646_ + 7.18 × OD_663_) × V/w, and *Car* (mg·g^−1^) = [(1000 × OD_470_ − 3.27 × Chl *a* – 104 × Chl *b*)/229] × V/w. V represents the total extract volume (L) and w is the fresh weight (g) of plant material.

#### 4.4.2. Photosynthetic Gas Exchange Parameter

On sunny, cloudless days between 9:00 and 11:30, functional leaves from the upper parts of the plants were selected for gas exchange measurements using a portable photosynthesis instrument (CIRAS-3, PP-Systems, Hitchin, UK). The net photosynthetic rate (P_n_), transpiration rate (T_r_), stomatal conductance (G_s_), intercellular CO_2_ concentration (C_i_), and leaf surface vapor pressure deficit (VPD) were measured. Stomatal limitation (L_s_), water-use efficiency (WUE), and CO_2_-use efficiency (CUE) were calculated according to the method described by Yang et al. [42]. During the measurements, the leaf chamber temperature was maintained at 25 °C, the photosynthetically active radiation was set at 1000 µmol·m^−2^·s^−1^, while the CO_2_ concentration was controlled between 380–420 µmol·mol^−1^.

#### 4.4.3. Chlorophyll *a* Fluorescence Parameter

Leaves used for gas exchange parameter measurements were dark-adapted for 20 min. The chlorophyll *a* fluorescence parameter was measured using a continuous excitation fluorometer (Handy PEA, Hansatech Instruments Limited, King’s Lynn, UK). The initial fluorescence (F_o_), maximum fluorescence yield in the dark-adapted state (F_m_), variable fluorescence (F_v_), electron transfer through PS Ⅱ (F_m_/F_o_), maximum photochemical quantum yield of PSII (F_v_/F_m_), potential photochemical activity (F_v_/F_o_), net closing rate of the reaction center at 100 µs (dVG/dt_o_), net closing rate of the reaction center at 300 µs (dV/dt_o_), absorbed light energy per reaction center (ABS/RC), heat dissipation per reaction center (DI_o_/RC), trapped energy flux per reaction center (TR_o_/RC), electron transport flux per reaction center (ET_o_/RC), energy transmitted to the end of the electronic chain per reaction center (RE_o_/RC), absorbed light energy per unit area (ABS/CS_o_), heat dissipation per unit area (DI_o_/CS_o_), trapped energy flux per unit area (TR_o_/CS_o_), electron transport flux per unit area (ET_o_/CS_o_), energy transmitted to the end of the electronic chain per unit area (RE_o_/CS_o_), and photosynthetic performance index (PI_abs_) were measured.

### 4.5. Measurement of Photosynthetic Carbon Metabolism

#### 4.5.1. Soluble Sugar Content

Soluble sugar content was determined using the phenol–sulfuric acid method [43] with sucrose as the standard. A standard curve was established using sucrose solutions (0–100 μg) for quantification. Briefly, a 0.3 g sample of leaves was placed in 10 mL of distilled water and boiled for 60 min. The extract was filtered and made up to 25 mL. A 0.5 mL aliquot of the sample solution was mixed with 1.5 mL of distilled water, followed by the addition of 1 mL of 9% phenol solution and 5 mL of concentrated sulfuric acid. The mixture was shaken and incubated for 30 min. The absorbance was measured at 485 nm using a UV–Visible Spectrophotometer (Lambda 365, PerkinElmer, Waltham, MA, USA). Soluble sugar content was calculated based on the standard curve, with results expressed in %.

#### 4.5.2. Sucrose, Fructose, and Glucose Contents

Sucrose, fructose, and glucose contents were quantified using commercial assay kits from Nanjing Jiancheng Bioengineering Institute (Nanjing, China). Specifically, sucrose content was measured using the Sucrose Measurement Kit (Cat# A099-1-1), with results expressed in μmol·g^−1^. Fructose content was determined using the Fructose Assay Kit (Cat# A085-1-1), reported in mg·g^−1^. Glucose content was analyzed via the Glucose Kit (glucose oxidase method, Cat# A154-1-1), expressed in mmol·g^−1^. All assays were performed according to the manufacturer’s protocols.

#### 4.5.3. SUS and SPS Activities

The method described by Zhang et al. [44] was as follows: A 0.3 g sample of leaves was ground in 3 mL of pre-chilled buffer (containing 50 mM HEPES-NaOH at pH 7.5, 50 mM MgCl_2_, 2 mM EDTA, 0.2% BSA, and 2% PVPP) then centrifuged at 4 °C. The supernatant was used as a crude enzyme solution. A 90 µL aliquot of the crude enzyme solution was mixed with 110 µL of the reaction system (containing 20 µL of 50 mM MgCl_2_, 50 µL of HEPES-NaOH at pH 7.5, 20 µL of 100 mM uridine diphosphate glucose, and 20 µL of 100 mM fructose). The mixture was incubated at 30 °C for 30 min then terminated by adding 0.2 mL of 2 M NaOH solution by boiling at 100 °C for 10 min. After cooling, absorbance was measured at 480 nm. The sucrose content after the enzyme reaction was calculated based on a standard curve. Simultaneously, a 90 µL aliquot of the crude enzyme solution was boiled at 100 °C for 10 min, then mixed with 0.2 mL of 2 M NaOH and the reaction system as described above. After cooling, the absorbance was measured at 480 nm to calculate the sucrose content of the extract before the enzymatic reaction. In the SUS reaction system, 100 mM fructose-6-phosphate was used instead of 100 mM fructose, and all other steps remained unchanged. The difference in the sucrose content before and after the enzymatic reaction was attributed to the sucrose formed by SPS. The activities of SUS and SPS were expressed as milligrams of sucrose per gram of fresh leaf weight per hour (mg·g^−1^·h^−1^).

#### 4.5.4. RuBisCO, GAPDH, and FBPase Activities

A 0.3 g sample of leaves was ground in 3 mL of PBS buffer (0.01 mM, pH 7.4) in a pre-chilled mortar. The homogenate was centrifuged at 13,000× *g* for 1 min at 4 °C, and the supernatant was collected to determine the RuBisCO, GAPDH, and FBPase activities. A plant enzyme-linked immunosorbent assay kit (Enzyme-linked Biotech, Shanghai, China) was used for measurements. RuBisCO, GAPDH, and FBPase activities were expressed in units per liter (U·L^−1^), where one unit (U) is defined as the amount of enzyme that catalyzes the conversion of 1 micromole of substrate per minute under the assay conditions.

#### 4.5.5. Gene Expression

RNA was extracted from *P. cordata* leaves treated with different Cd concentrations using a Plant RNA Extraction Kit (Biofit Biotech, Chengdu, China). The concentration and purity of RNA were determined using a Thermo NanoDrop 2000 microspectrophotometer (Thermo Fisher Scientific, Waltham, MA, USA). RNA integrity was assessed using 1% agarose gel electrophoresis at 130 V and 140 mA for 20 min. Reverse transcription was performed using TransScript^®^ One-Step gDNA Removal and cDNA Synthesis SuperMix (TransGen Biotech, Beijing, China).

The protein sequences of *Arabidopsis thaliana* SUS, SPS, GAPDH, and FBPase genes were aligned with the transcriptome protein sequences of *P. cordata* to identify the most homologous gene sequences for expression analysis. The RuBisCO large subunit gene (*RBCL*), which encodes the large subunit of RuBisCO, is important in the chloroplast genome of plant cells. The *RBCL* gene sequence was obtained from the NCBI for Biotechnology Information public database (Gene ID: 41954708). PCR primers were designed using the Premier 5 software (Premier Biosoft, Palo Alto, CA, USA). The gene specificity of each primer was confirmed using a BLAST search of public databases (National Center for Biotechnology Information, Bethesda, MD, USA), and the results are provided in Table S1. The TFE gene from *P. cordata* was selected as the internal reference gene [19]. qRT-PCR was performed using PerfectStart^®^ Green qPCR SuperMix (TransGen Biotech, Beijing, China). Each qRT-PCR analysis included three biological and three technical replicates.

### 4.6. Statistical Analyses

All statistical analyses were performed using GraphPad Prism v9.5.1 and SPSS v27.0.1. The normality of data distribution was first verified by Shapiro–Wilk tests (*p* > 0.05), followed by assessment of homogeneity of variance using Levene’s test (*p* > 0.05). Only datasets satisfying both assumptions were analyzed by one-way ANOVA with the Tukey’s multiple comparisons test to assess the statistical significance. The results are presented as mean ± standard error (SE) of triplicate biological replicates unless otherwise specified, with a significance threshold of *p* ≤ 0.05 applied uniformly. Graphical representations were generated using GraphPad Prism v9.5.1 and finalized in Adobe Illustrator 2024.

## 5. Conclusions

**Figure 8:**
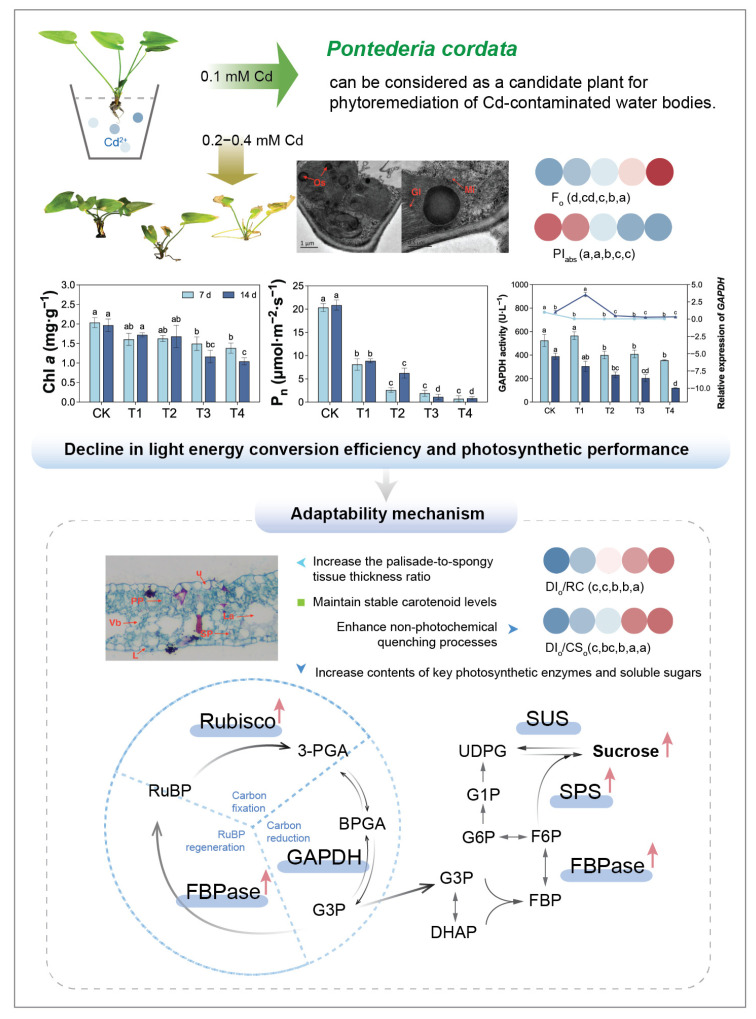
Photosynthetic physiological responses of *Pontederia cordata* to Cd stress. Note: The applied Cd concentrations (0.1–0.4 mM, equivalent to 11.2–44.8 mg·L^−1^) are comparable to those reported in heavily contaminated aquatic environments (0.7–40 mg·L^−1^ Cd) and significantly exceed typical background concentrations (<0.01 mg·L^−1^) in uncontaminated waters [45]. Different lowercase letters per day indicate significant differences among treatments (one-way ANOVA, Tukey’s test, *p* ≤ 0.05).

*Pontederia cordata* demonstrated concentration-dependent physiological responses and adaptive mechanisms under Cd stress (Figure 8). At 0.1 mM Cd, plants maintained normal growth with an intact PSII structure and function and chloroplast ultrastructure, showing no toxic symptoms. However, 0.2–0.4 mM Cd treatments disrupted chloroplast integrity, decreased chlorophyll content, and suppressed GADPH expression or activity, ultimately impairing light energy conversion efficiency and photosynthetic performance. In response, *P. cordata* adopts a coordinated set of adaptive strategies, including anatomical adjustments (such as maintaining palisade tissue thickness and increasing the palisade-to-spongy tissue thickness ratio), the stabilization of carotenoid levels, enhancement of photoprotective mechanisms (non-photochemical quenching), elevating levels of key photosynthetic enzymes (RuBisCO and FBPase), and the accumulation of soluble sugars (sucrose, glucose, and fructose) as metabolic reserves.

## Data Availability

Data are contained within the article.

## Supplementary Materials

The following supporting information can be downloaded at: https://www.mdpi.com/article/10.3390/plants14091344/s1, Table S1. Fluorescence quantitative PCR primer sequences of gene and reference gene; Figure S1. Morphological changes of *P. cordata* exposed to different Cd^2+^ concentrations for 7 d and 14 d; Table S2. Damage levels of *P. cordata* exposed to different Cd^2+^ concentrations; Table S3. Changes of Chlorophyll *a* fluorescence parameters in leaves of *P. cordata* exposed to different Cd^2+^ concentrations for 7 d and 14 d. Different lowercase letters on the same line indicate significant differences among treatments (*p* < 0.05).
