# Supplementary material for: Photosynthetic Responses of Pontederia cordata to Cadmium Stress: Anatomical Structure, Ultrastructure, Physiology, and Gene Expression
Source: Plants (Basel). 2025 Apr 29;14(9):1344. doi: 10.3390/plants14091344 (PMC12073675; doi:10.3390/plants14091344)
Supplement: Supplementary file 1 [file plants-14-01344-s001.zip › plants-3565989-supplementary.pdf]

Table S1: Fluorescence quantitative PCR primer sequences of gene and reference gene

Gene	Primer（5'-3'）
SUS	F：GATGCTGAAGATGGATTTTAACCT
	R：CAACAAAGGAAAGACTGGGTGAT
SPS	F：ATGAAAAATGCCCGACACTAGA
	R：GCCGTGCGAAACTATTCTCTAC
RBCL	F：GCCCACCTCACGGTATCCA
	R：GGTTTCGGCTTGCGCTTT
GAPDH	F：GCGCTAGAAGGGTCTGGATTA
	R：GGTGATTTGATGATTCTGTGTCTTT
FBP	F：GTAACAGCTATTAAACAAGGGGTGC
	R：CACCACTGTGGCAATCTGTCAT
TFE1	F：CACTGTTTTCTGCTGCCCTT
	R：CACTGTTTTCTGCTGCCCTT

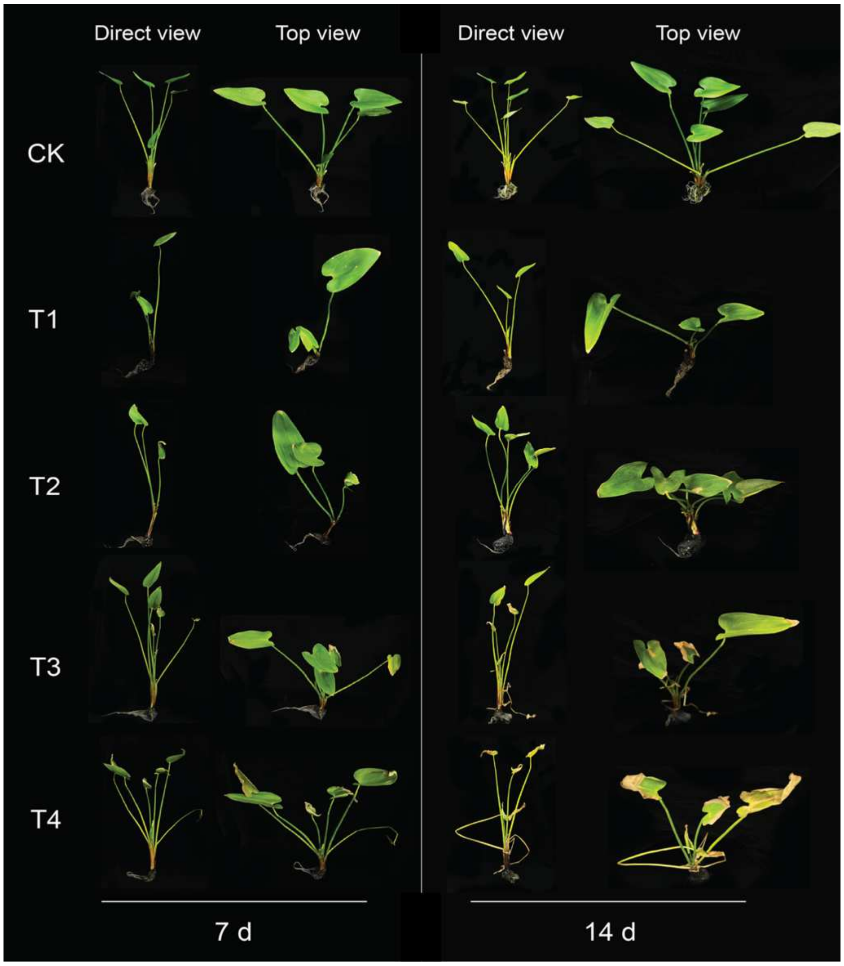

Figure S1. Morphological changes of *P. cordata* exposed to different Cd^{2+} concentrations for 7 d and 14 d

Table S2. Damage levels of *P. cordata* exposed to different Cd^{2+} concentrations

Date	Treatments	Plant morphology	
		Damage symptoms	Damage levels
7 d	CK	No stress-induced symptoms	0
	T1	No stress-induced symptoms	0
	T2	Minor yellowing of leaf tips, margins, or veins in some plants	1
	T3	Severe browning of leaf tips and margins in most plants	2
	T4	Leaf wilting, abscission, or even death in some plants	3
14 d	CK	No stress-induced symptoms	0
	T1	No stress-induced symptoms	0
	T2	Minor yellowing of leaf tips, margins, or veins in some plants	1
	T3	Leaf wilting, abscission, or even death in some plants	3
	T4	Leaf wilting, abscission, or even death in most plants	4

Note: Based on the degree of Cd toxicity to leaves, the plant damage index was divided into six levels (0, 1, 2, 3, 4, 5), with level 5 indicating complete plant death.

Table S3. Changes of Chlorophyll *a* fluorescence parameters in leaves of *P. cordata* exposed to different Cd^{2+} concentrations for 7 d and 14 d. Different lowercase letters on the same line indicate significant differences among treatments ($P < 0.05$).

Index	Date	Treatments				
		CK	T1	T2	T3	T4
F_o	7 d	512.67±13.91b	646.67±71.10ab	759.00±102.77a	634.67±50.84ab	698.00±8.08ab
	14 d	463.00±11.36d	494.33±9.61cd	536.67±20.84c	594.67±16.56b	723.67±16.33a
F_m	7 d	2554.00±104.40ab	2828.00±79.41a	2474.50±94.97b	2422.33±130.23b	2269.00±9.81b
	14 d	2846.67±41.87a	2743.00±227.01a	2791.00±54.54a	2914.33±174.22a	3154.00±57.71a
F_v	7 d	2041.33±94.43ab	2181.33±53.24a	1715.50±197.74bc	1787.67±131.85bc	1571.00±17.90c
	14 d	2383.67±31.55a	2248.67±224.60a	2254.33±33.75a	2086.33±113.34a	2211.33±87.42a
F_m/F_o	7 d	4.98±0.13a	4.46±0.43ab	3.42±0.60b	3.87±0.37ab	3.25±0.05b
	14 d	6.15±0.07a	5.55±0.45ab	5.21±0.10abc	4.95±0.48bc	4.36±0.10c
F_v/F_m	7 d	0.80±0.01a	0.77±0.02ab	0.69±0.05ab	0.74±0.02ab	0.69±0.00b
	14 d	0.84±0.00a	0.82±0.02a	0.81±0.00a	0.72±0.04b	0.70±0.04b
F_v/F_o	7 d	3.98±0.13a	3.47±0.43a	2.50±0.60ab	2.87±0.37ab	2.25±0.05b
	14 d	5.15±0.08a	4.88±0.15a	4.21±0.10b	3.52±0.12c	3.27±0.05c
dVG/dt_o	7 d	0.51±0.01c	0.94±0.13b	1.18±0.07ab	0.95±0.13b	1.39±0.12a
	14 d	0.51±0.02c	0.63±0.06c	0.84±0.02b	0.95±0.04b	1.10±0.05a
dV/dt_o	7 d	0.85±0.05c	1.24±0.10b	1.41±0.04ab	1.27±0.11b	1.61±0.04a
	14 d	0.86±0.02c	0.97±0.07c	1.21±0.02b	1.30±0.03b	1.52±0.10a
ABS/RC	7 d	2.57±0.03c	3.23±0.22b	3.87±0.30ab	3.64±0.13ab	4.03±0.24a

	14 d	2.38±0.05b	2.62±0.10b	3.11±0.08a	3.30±0.10a	3.17±0.09a
DI_o/RC	7 d	0.52±0.02b	0.74±0.11ab	1.26±0.30a	0.88±0.16ab	1.24±0.09a
	14 d	0.39±0.01c	0.48±0.06c	0.60±0.03b	0.68±0.03b	0.73±0.02a
TR_o/RC	7 d	2.05±0.02c	2.48±0.11ab	2.61±0.00ab	2.42±0.14b	2.78±0.15a
	14 d	1.99±0.03c	2.14±0.05c	2.51±0.05ab	2.42±0.06b	2.65±0.05a
ET_o/RC	7 d	1.20±0.06a	1.25±0.08a	1.20±0.07a	1.15±0.06a	1.17±0.19a
	14 d	1.13±0.02b	1.17±0.03ab	1.30±0.04ab	1.32±0.10a	1.17±0.01ab
RE_o/RC	7 d	0.61±0.06a	0.43±0.07bc	0.35±0.03c	0.45±0.11bc	0.52±0.02ab
	14 d	0.47±0.03a	0.43±0.03ab	0.54±0.01a	0.35±0.05b	0.32±0.04b
ABS/CS_o	7 d	512.67±13.91b	646.67±71.10ab	759.00±102.77a	634.67±50.84ab	698.00±8.08ab
	14 d	463.00±11.36d	494.33±9.61cd	536.67±20.84c	594.67±16.56b	723.67±16.33a
DI_o/CS_o	7 d	103.06±3.71b	150.36±29.19ab	253.99±72.79a	169.01±25.84ab	214.88±5.90ab
	14 d	75.33±2.68c	90.33±7.73bc	103.27±5.96b	141.66±2.87a	146.51±7.64a
TR_o/CS_o	7 d	409.61±11.74b	496.30±42.35ab	505.01±29.98a	465.66±28.44ab	483.12±2.18ab
	14 d	387.67±8.72b	404.01±11.49b	433.40±14.88ab	478.25±11.69a	478.51±34.35a
ET_o/CS_o	7 d	240.00±13.44a	247.81±13.34a	231.07±6.17ab	221.50±9.54ab	202.29±9.34b
	14 d	220.20±4.15a	221.64±14.10a	224.84±9.13a	214.67±5.42a	222.01±12.34a
RE_o/CS_o	7 d	108.85±18.80a	85.92±6.53ab	66.12±0.93b	85.18±9.60ab	90.42±2.56ab
	14 d	92.52±5.48a	80.02±4.25b	92.74±3.02a	70.24±1.34b	81.63±0.94ab
PI_{abs}	7 d	2.20±0.17a	1.92±0.08a	1.13±0.07b	0.62±0.05c	0.40±0.00c
	14 d	2.86±0.12a	2.62±0.17a	1.46±0.07b	0.90±0.05c	0.80±0.03c
